# Development of conventional and real-time multiplex PCR-based assays for estimation of natural infection rates and *Trypanosoma cruzi* load in triatomine vectors

**DOI:** 10.1186/s13071-017-2343-x

**Published:** 2017-08-29

**Authors:** Otacilio C. Moreira, Thaiane Verly, Paula Finamore-Araujo, Suzete A. O. Gomes, Catarina M. Lopes, Danielle M. de Sousa, Lívia R. Azevedo, Fabio F. da Mota, Claudia M. d’Avila-Levy, Jacenir R. Santos-Mallet, Constança Britto

**Affiliations:** 1 Laboratório de Biologia Molecular e Doenças Endêmicas, Instituto Oswaldo Cruz/Fiocruz, Rio de Janeiro, Brazil; 2 Laboratório de Biodiversidade de Parasitas e Vetores, Universidade Federal Fluminense/UFF, Rio de Janeiro, Niterói Brazil; 3 Laboratório Interdisciplinar de Vigilância Entomológica de Diptera e Hemiptera, Instituto Oswaldo Cruz/Fiocruz, Rio de Janeiro, Brazil; 4 Laboratório de Biologia Computacional e Sistemas, Instituto Oswaldo Cruz/Fiocruz, Rio de Janeiro, Brazil; 5 Laboratório de Estudos Integrados em Protozoologia, Instituto Oswaldo Cruz/Fiocruz, Rio de Janeiro, Brazil

**Keywords:** *Trypanosoma cruzi*, Multiplex PCR, Real-time qPCR, Triatomines, Natural infection, Parasite load

## Abstract

**Background:**

Chagas disease is a complex anthropozoonosis with distinct domestic and sylvatic mammal species acting as potential reservoirs. The diversity of vector species and their habitats are among the factors that hinder the control of the disease. Control programs periodically monitor the prevalence of *T. cruzi* infection in insect bugs through microscopical observation of diluted feces. However, microscopy presents limited sensitivity in samples with low parasite numbers, difficulties in examining all evolutionary stages of the insect and may in turn be limited to differentiate *T. cruzi* from other morphologically similar trypanosomatids. Here, we report two highly sensitive and accurate methodologies to infer *T. cruzi* infection rates and to quantify parasite load in the gut of field-collected triatomines.

**Methods:**

Triatomines were manually collected in the period 2011–2012 and 2014–2015, in domestic, peridomestic or sylvatic habitats in rural areas of 26 municipalities, encompassing three distinct Brazilian biomes: Caatinga, Cerrado and Atlantic Rainforest. Following morphological and taxonomical identification, the search for flagellated protozoa was performed by optical microscopy. A conventional PCR targeting *T. cruzi* kDNA and a *Taq*Man qPCR directed to the parasite nuclear satellite DNA (SAT) were developed, both in multiplex, with the triatomine 12S subunit ribosomal RNA gene, used as internal amplification control. Both methods were used for detection (kDNA-PCR) and parasite load quantification (SAT-DNA-qPCR), to investigate *T. cruzi* infection in captured triatomines.

**Results:**

The combined methods were assayed on a panel of 205 field-collected triatomine samples. Diagnostic analysis revealed 21% positivity for the kDNA-PCR, whereas microscopic examination enabled identification of *T. cruzi* in only 7.0% of the PCR-positive samples. Negative PCR results were confirmed by the absence of *T. cruzi* flagellates using microscopy. Caatinga biome yielded the highest *T. cruzi* infection rate (60%), followed by the Atlantic Rainforest and Cerrado with 7.1 and 6.1%, respectively. In addition, a wide range distribution of parasite load, varying from 8.05 × 10^-2^ to 6.31 × 10^10^ was observed with a median of 2.29 × 10^3^ *T. cruzi*/intestine units. When parasite load was analyzed by triatomine species, a significantly higher median was found for *Panstrongylus lutzi* in comparison with *Triatoma brasiliensis*.

**Conclusions:**

Our results demonstrate highly sensitive PCR-based methodologies to monitor *T. cruzi* infection in triatomines. In addition, the qPCR assay offers the possibility of further evaluation parasite load, as a promising biomarker of the vectorial capacity of triatomines in Chagas disease endemic areas.

**Electronic supplementary material:**

The online version of this article (10.1186/s13071-017-2343-x) contains supplementary material, which is available to authorized users.

## Background

Chagas disease (ChD) is an important neglected tropical illness caused by the flagellate protozoan *Trypanosoma cruzi* (Kinetoplastida: Trypanosomatidae). The protozoan is primarily transmitted by insects belonging to the subfamily Triatominae (Hemiptera: Reduviidae), including 18 genera and 151 species [1]. The disease is a complex anthropozoonosis with distinct domestic and sylvatic mammal species acting as potential reservoirs of the infection. Although considered neglected, ChD is endemic in South and Central Americas, and in some regions of the USA [2]. The disease has globally expanded, with more than 5 million people infected and approximately 70 million living at risk [3], and is now reported in the Americas, Europe, Australia and Asia due to the migration of infected individuals from endemic parts of the world [4].

The classical transmission form to humans, via triatomine feces, is correlated to the presence of *T. cruzi* infected bugs in domestic and/or peridomestic areas. The diversity of vector species and their habitats are among the factors that hinder the control of the disease. The importance of three genera, *Triatoma*, *Rhodnius* and *Panstrongylus*, lies in the fact that some of their members feed on humans and synanthropic mammals and may thus transmit *T. cruzi* [5]. In Brazil, four triatomine species play a direct role in the epidemiology of the disease: *Triatoma brasiliensis* Neiva, 1911; *Panstrongylus megistus* (Burmeister, 1835); *Triatoma pseudomaculata* Corrêa & Espínola, 1964 and *Triatoma sordida* (Stål, 1859). These species are found in sylvatic habitats despite being adapted to domestic and peridomestic areas. Other species identified in wild ecotopes, such as *Triatoma costalimai* Verano & Galvão, 1958; *Triatoma wygodzinskyi* Lent, 1951; *Rhodnius neglectus* Lent, 1954 and *Triatoma rubrovaria* (Blanchard, 1843), maintain an enzootic cycle involving wild mammals in a variety of terrestrial or arboreal biotopes [6]. In addition, *Rhodnius brethesi* Matta, 1919 has been reported as an active vector in the Amazon Region, considering the rates of *T. cruzi* transmission to people involved in plant extraction activities [7].

The risk of *T. cruzi* transmission in endemic areas mainly depends on the density of triatomine bugs and the prevalence of *T. cruzi* infection in insect vectors, humans and animal reservoirs [8]. The impact in reducing transmission by the vectors *Triatoma infestans* (Klug, 1834) and *Rhodnius prolixus* (Stål, 1859) has been observed particularly in the Southern Cone countries of South America and in Central America, respectively [9, 10]. Transmission prevention programs include insecticide-based campaigns, housing improvements, health education and blood donor screening [9]. Yet, the wide use of insecticides has created resistant triatomine populations [11–13].

The screening of *T. cruzi* infection in field-collected triatomines from different regions of Brazil is relevant for a better understanding of ChD epidemiology and for giving support to developing programs to control disease spreading. Furthermore, information regarding *T. cruzi* parasite load in triatomines could bring an important contribution to infer potential vectorial capacity of distinct species. Control programs periodically monitor the prevalence of *T. cruzi* infection in insect bugs through optical microscopy observation of diluted feces in the search for active trypanosomes. Although this method is inexpensive and widely used, it presents limited sensitivity in samples with low parasite numbers [14], difficulties in examining all evolutionary stages of the insect and may in turn be limited to differentiate *T. cruzi* from other morphologically similar trypanosomatids. In addition, it is a laborious and time-consuming procedure, where fresh insect examination is needed to detect live parasites [15].

Our group reported, for the first time, a standardized and accurate SYBR-Green qPCR assay to quantify parasite load levels in *R. prolixus* midgut and rectum, to investigate the role of cruzipain in the interaction of *T. cruzi* with the triatomine host [16]. In the present study, we propose a combination of two multiplex PCR-based assays (conventional and *Taq*Man real-time qPCR), as molecular diagnostic tools for the accurate detection and quantification of *T. cruzi* DNA in triatomines. The conventional PCR (qualitative) and qPCR (quantitative) were based on the parasite targets kinetoplast DNA or kDNA [17] and nuclear satellite DNA [18], respectively, together with the triatomine 12S subunit ribosomal RNA gene, as an internal control, in both assays. The generated data could bring more insight to investigate the association between PCR positivity and parasite load in field-collected triatomines and the risk for *T. cruzi* transmission to humans in endemic areas of Chagas disease.

## Methods

### Parasite culture and *R. prolixus* maintenance

*Trypanosoma cruzi* CL Brener (COLPROT 005), Dm28c (COLPROT 0010), INPA 4167 (COLPROT 0607) and *Trypanosoma rangeli* (Macias strain - COLPROT 0273) were obtained from the Coleção de Protozoários da Fundação Oswaldo Cruz, Rio de Janeiro, Brazil (Fiocruz, COLPROT, http://www.colprot.fiocruz.br). Epimastigote forms were grown in 3.7% BHI medium, containing 0.002% hemin and supplemented with 10% heat-inactivated FBS, at 28 °C for 4 days to reach late-log growth phase. Parasites were harvested by centrifugation (1500× *g* for 5 min at 25 °C), washed three times with 0.15 M NaCl, 0.01 M phosphate buffer pH 7.2 (PBS), prior to DNA extraction.

*Rhodnius prolixus* specimens were reared and maintained with controlled temperature and humidity conditions, as previously described [19]. The insects were obtained from the insectary of the Laboratório de Bioquímica e Fisiologia de Insetos, Instituto Oswaldo Cruz (Fiocruz), Rio de Janeiro, Brazil.

### Field-collected triatomines

**Fig. 1:**
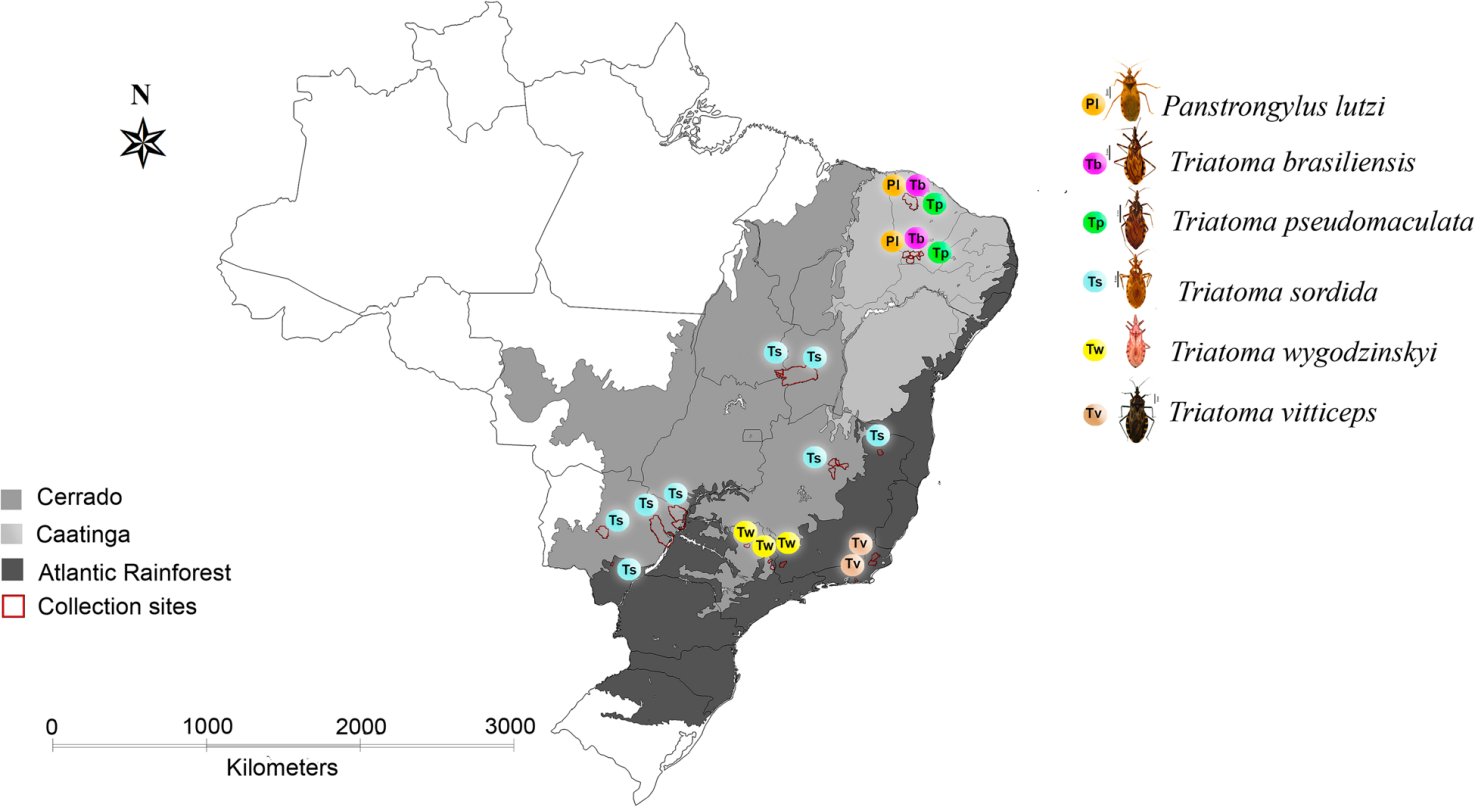
Representative map of Brazil indicating the distribution of triatomine collection sites and the studied biomes

Insects were captured in the period 2011–2012 and 2014–2015, in 26 municipalities distributed in three Brazilian biomes (Caatinga, Cerrado and Atlantic Rainforest), where each one constitutes idiosyncratic landscape mosaics (Fig. 1). Caatinga, a biome of northeastern Brazil, is characterized by a semiarid climate with vegetation represented by a mosaic of xerophytic, deciduous, thorny scrub, with tree coverage lower than 60%. Cerrado biome is a heterogeneous, floristic savannah that covers more than 2 million km^2^ and extends from central Brazil to parts of Bolivia and Paraguay, representing the second largest South American biome. Atlantic Rainforest is formed by a set of forest formations and associated ecosystems such as salt marshes, mangroves and high fields, occupying an area *c.*13% of the country [20].

Householders were informed of the aim of the research project, anticipated benefits and potential participation. Each environment was georeferenced and potential ecotopes, including dwellings and rock-piles, under tree bark and nests of rodents or birds, were investigated for the presence of triatomines. Manual collections with the use of forceps were carried out in both wild and dwellings sites. All captured bugs were placed in labelled plastic bags with folded filter paper inside and transported to the Laboratório Interdisciplinar de Vigilância Entomológica de Diptera e Hemiptera, Instituto Oswaldo Cruz (Fiocruz), Rio de Janeiro, Brazil, where they were identified to species [21, 22] and stage and collection site, followed by the search of flagellated protozoa by optical microscopy. Nymph instars were classified according to the identification of the adult stages found in the colony.

### Optical microscopy

All live third-, fourth- and fifth-instar nymphs and adult triatomines were examined for *T. cruzi* infection by microscopic examination (ME) of fecal samples. One fecal drop from each live bug, obtained by abdominal compression, was diluted in one drop of sterile saline solution, NaCl 0.85% (*c.*50 μl) on a glass slide and examined fresh by microscopy, for active trypanosomes, at magnifications of 200–400×. For dead insects, midgut and rectum were dissected and soaked in a sterile Petri dish containing 2 ml saline solution. The homogenate (50 μl) was observed between slide and coverslip for *T. cruzi* search by optical microscopy. Forceps were rinsed in 10% bleach and 70% ethanol between extracting successive samples.

### DNA extraction for molecular assays

Following ME, live insects used for molecular studies were placed in absolute ethanol until being dissected under a stereomicroscope to collect midgut and rectum. For dead insects, the intestinal content homogenates previously collected for ME were stored in sterile microtubes at 4 °C for DNA extraction. Non-infected *R. prolixus* specimens maintained in insectary were used as negative controls during DNA extraction of field-collected triatomines. Samples were pretreated for 2 h at 56 °C with 50 μl lysis buffer containing 10 mM Tris-HCl (pH 9.2), 1 mM EDTA and 150 μg/ml proteinase K (Sigma-Aldrich, St. Louis, MO, USA). After treatment, samples were centrifuged (9800× *g*, 15 min, 4 °C) and the supernatant was collected and diluted in TE buffer (1 mM Tris-HCl pH 9.2, 1 mM EDTA) to reach 200 μl total volume. DNA was purified from the lysate using the QIAamp DNA mini kit (Qiagen, Hilden, Germany) with slight modifications [23]. Briefly, at the elution step, 100 μl AE buffer were added to the silica-membrane column and maintained at room temperature for 10 min, before DNA elution. DNAs were stored at -20 °C until use, and their purity and concentration were determined using a Nanodrop 2000c spectrophotometer (Thermo Scientific, Waltham, MA, USA) at 260/280 and 260/230 nm.

### Multiplex conventional PCR (cPCR)

Conventional PCR assays were carried out in a final volume of 50 μl, containing: 5 μl DNA (20–25 ng), 5 μl 10× *Taq* Platinum buffer, 0.2 mM dNTPs, 4.5 mM MgCl_2_, 1.25 U *Taq* Platinum DNA polymerase (Life Technologies, Carlsbad, CA, USA), 200 nM 121/122 primers (*T. cruzi* kDNA) [17, 24] and 100 nM P2B/P6R primers (triatomine 12S rRNA gene). *Trypanosoma cruzi* and triatomine primers are described in Table 1. Amplifications were performed in the GeneAmp PCR System 9700 (Life Technologies), as follows: 94 °C for 12 min; 36 cycles at 94 °C for 30 s, 55 °C for 30 s and 72 °C for 30 s, with a final extension at 72 °C for 10 min. PCR products were loaded onto 2% (*w*/*v*) agarose gels and submitted to electrophoresis at 80 V for 40 min. Gels were stained with a Nancy-520 fluorescent dye (Sigma-Aldrich). This procedure allows for discrimination between *T. cruzi* and *T. rangeli*, and each PCR reaction was run with a positive control for *T. cruzi* and *T. rangeli* epimastigotes, and a reagent negative control (without DNA). In addition, a PCR multiplex positive control formed by mixing 1:1 DNA from one dissected midgut of non-infected adult *R*. *prolixus* with DNA of *T. cruzi* CL Brener-epimastigotes (10^2^ cells) was used. The sensitivity and specificity of microscopy for detecting *T. cruzi* infection in triatomines were calculated using PCR as the gold standard.

**Table 1:** Primer and probe sequences for cPCR and qPCR assays

Target	Primer/Probes	Amplicon size (bp)	Sequence (5′–3′)	Reference
*T. cruzi* kinetoplast DNA (kDNA)	121 (Forward)	330	AAATAATGTACGGG(T/G)GAGATGCATGA	[17, 24]
	122 (Reverse)		GGTTCGATTGGGGTTGGTGA ATATA	
*T. cruzi s*atellite DNA (SAT-DNA)	Cruzi 1 (Forward)	165	ASTCGGCTGATCGTTTTCGA	[18]
	Cruzi 2 (Reverse)		AATTCCTCCAAGCAGCGGATA	
	Cruzi 3 (Probe)		FAM-TTGGTGTCCAGTGTGTG-NFQ-MGB	
Triatomine 12S rRNA	P2B (Forward)	163	AAAGAATTTGGCGGTAATTTAGTCT	Present study
	P6R (Reverse)		GCTGCACCTTGACCTGACATT	
	Triat (Probe)		VIC-TCAGAGGAATCTGCCCTGTA-NFQ-MGB	

### Quantitative multiplex real-time PCR (qPCR)

The qPCR reactions were carried out in a final volume of 20 μl, containing 2 μl DNA (8–10 ng), 2× *Taq*Man® Universal PCR Master Mix (Applied Biosystems, Foster City, CA, USA), 600 nM cruzi1/cruzi2 primers and 250 nM Cruzi3 probe (FAM/NFQ-MGB) targeting *T. cruzi* nuclear satellite DNA (SAT-DNA), 300 nM P2B primer, 500 nM P6R primer and 150 nM Triat Probe (VIC/NFQ-MGB) (Applied Biosystems) directed to the 12S ribosomal subunit gene of triatomines. Sequences of both sets of primers and probes are presented in Table 1. The cycling conditions were as follows: 50 °C for 2 min, 95 °C for 10 min, followed by 40 cycles at 95 °C for 15 s and 58 °C for 1 min. Amplifications were performed in the ABI Prism 7500 Fast (Applied Biosystems). Standard calibration curves for *T. cruzi* and triatomine targets were constructed by serially diluting total DNA obtained from non-infected triatomine intestine samples (adults *R*. *prolixus* from insectary) spiked with 10^5^ *T. cruzi* epimastigotes (Dm28c clone, TcI). The resulting DNA was serially diluted to a range of 10^5^ to 0.5 *T. cruzi* equivalents and 5 to 0.002 triatomine intestine unit equivalents.

To determine the limit of detection (LOD) for *T. cruzi* DNA in triatomines, intestine samples were spiked with 20 to 0.00128 parasite equivalents and assayed in 12 replicates for three consecutive days. The LOD was calculated as the lowest parasitic load corresponding to ≥ 95% of PCR positive results, according to clinical and laboratory standard guidelines [25].

### Statistical analysis

All qPCR assays were performed in experimental duplicate. Results were expressed as the mean of *T. cruzi*/intestine units. Statistical analysis of parasite load between triatomine species (Student’s t-test or Mann-Whitney rank sum test, and Analysis of Variance, ANOVA) were performed with SigmaPlot v12.0 software (Systat Software, Inc). The LOD of the real-time qPCR was determined by Probit regression analysis, using Minitab 15 Statistical Software (Minitab Inc., State College, PA).

## Results

The main goal of this study is the development of PCR approaches to infer *T. cruzi* infection rates and parasite load in field-collected triatomines from Chagas disease endemic areas. A conventional multiplex PCR (cPCR) was developed targeting *T. cruzi* kinetoplast DNA minicircles (kDNA) and triatomine 12S rRNA gene, used as an internal amplification control, for the screening of *T. cruzi* DNA in triatomine samples and enabling its differentiation from *T. rangeli*. Further, we propose a quantitative *Taq*Man-based multiplex real-time PCR (qPCR), targeting *T. cruzi* nuclear satellite DNA (SAT-DNA - FAM/NFQ-MGB Probe) and triatomine 12S rRNA gene (VIC/NFQ-MGB Probe), to estimate the normalized parasite load, according to the DNA amount recovered from field-collected triatomine samples.

**Fig. 2:**
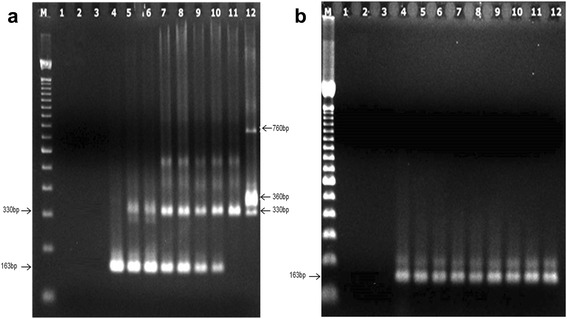
Assaying distinct *T. cruzi* strains/clones and triatomine species in the conventional multiplex PCR. **a** Reconstituted DNA samples (*R*. *prolixus* + *T. cruzi* DNAs) were used for the detection of distinct *T. cruzi* strains/clones with 121/122 primers (kDNA) in the multiplex format. Lane M: 100 bp DNA molecular marker; Lanes 1, 2: negative PCR controls (PCR reagents without DNA and ultra-pure water, respectively); Lane 3: DNA extraction negative control (extraction reagents + ultra-pure water); Lane 4: non-infected triatomine control (*R. prolixus* from insectary); Lanes 5, 6: DNA from *R*. *prolixus* midgut mixed with *T. cruzi* CL Brener (TcVI) DNA; Lanes 7, 8: DNA from *R*. *prolixus* midgut mixed with *T. cruzi* Dm28c (TcI) DNA; Lanes 9, 10: DNA from *R*. *prolixus* midgut mixed with *T. cruzi* INPA 4167 (TcIV) DNA; Lane 11: *T. cruzi* positive control (DNA obtained from 10^2^ CL Brener epimastigotes); Lane 12: *T. rangeli* positive control (DNA obtained from 10^2^ Macias strain epimastigotes). **b** Detection of distinct triatomine species using P2B/P6R primers (12S rRNA gene). Five individual midguts of each species were used as DNA source. Lane M: 100 bp DNA molecular marker; Lanes 1, 2: negative PCR controls (PCR reagents without DNA and ultra-pure water, respectively); Lane 3: DNA extraction negative control (extraction reagents in ultrapure water); Lanes 4, 5: *R. neglectus*; Lanes 6, 7: *T. costalimai*; Lane 8: *T. pseudomaculata*; Lane 9: *T. wygodizinskyi*; Lane 10: *T. sordida*; Lanes 11, 12: *R. prolixus*

Taking into account the diversity of *T. cruzi* lineages and triatomine species circulating in Brazilian endemic areas, we first tested the multiplex cPCR with reconstituted samples consisted of DNA obtained from dissected midgut of *R*. *prolixus* specimens, maintained in insectary, mixed 1:1 with DNA extracted from 10^2^ *T. cruzi* epimastigotes of the strains/clones Dm28c (TcI), INPA 4167 (TcIV) and CL Brener (TcVI) (Fig. 2a). The amplification of the variable regions of minicircles kDNA was observed in all tested *T. cruzi* strains/clones (330 bp fragment) and *T. rangeli* (330, 360 and 760 bp fragments), allowing the differentiation between both species. In the duplex PCR, we amplified successfully the 12S rRNA gene of triatomines (163 bp) in the same samples. In parallel, the performance of the new primers designed for the subfamily Triatominae, used as an internal amplification control, was assayed using distinct triatomine species not infected with *T. cruzi*: *R. neglectus*, *T. costalimai*, *T. pseudomaculata*, *T. wygodizinskyi*, *T. sordida* and *R. prolixus* (Fig. 2b). The characteristic 163 bp product was revealed in all triatomine samples.

In the present investigation, 205 field-collected triatomines were used to assess the performance of two PCR-based strategies for detection and quantification of *T. cruzi* parasites directly from intestinal content. The insects were collected in domestic, peridomestic or sylvatic habitats in rural areas encompassing three distinct Brazilian biomes. Following species identification, the triatomines were sorted according to the respective biome (Fig. 1): Caatinga (*n* = 55): *T. pseudomaculata* (26/55; 47.4%), *T. brasiliensis* (22/55; 40.0%) and *P. lutzi* (7/55; 12.6%); Cerrado (*n* = 66): *T. sordida* (41/66; 62.1%) and *T. wygodizinskyi* (25/66; 37.9%); Atlantic Rainforest (*n* = 84): *T. sordida* (54/84; 64.3%), *T. wygodizinskyi* (23/84; 27.4%) and *Triatoma vitticeps* (7/84; 8.3%). Using manual collections, most of the specimens (74.7%) were captured in peridomestic ecotopes in the three biomes. Only four *T. brasiliensis* adults (1.9% of all captured insects) were found in the intradomicile environment, and all *T*. *wygodizinskyi* specimens (23.4% of the captured insects) were from sylvatic habitats, collected with the use of forceps on the cracks of rocks. A detailed description of the captured triatomines is presented (Additional file 1: Table S1).

### Parasitological and molecular diagnosis of field-collected triatomines

The diagnostic analysis to detect *T. cruzi* in the 205 field-collected triatomines revealed a kDNA-PCR positivity of 21% (43/205), and the flagellate was detected by optical microscopy in three out of the 43 PCR-positive samples (7.0%). All PCR-negative samples were also negative for the parasite presence by microscopy (Table 2).

**Table 2:** Diagnostic results of *T*. *cruzi* infection generated by conventional multiplex PCR (cPCR) and optical microscopy in field-collected triatomines. Conventional PCR (cPCR) was considered as gold standard

	Field triatomine samples (*n* = 205)	
	cPCR +	cPCR -
Microscopy +	3 (7%)	–
Microscopy -	40 (93%)	162 (100%)
Total	43	162

**Fig. 3:**
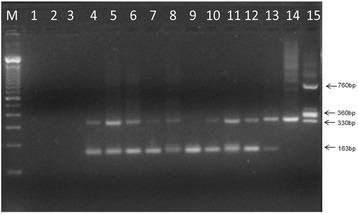
Representative gel for molecular detection of *T. cruzi* DNA in field triatomine samples by multiplex conventional PCR. Lane M: 100 bp molecular marker; Lanes 1, 2: PCR negative controls (PCR reagents without DNA and ultra-pure water, respectively); Lane 3: DNA extraction negative control (extraction reagents in ultrapure water); Lanes 4, 12: field triatomine samples; Lane 13: multiplex PCR positive control (*R. prolixus* midgut spiked with ~10^2^ CL Brener epimastigotes); Lane 14: *T. cruzi* positive control (DNA extracted from 10^2^ CL Brener epimastigotes); Lane 15: *T. rangeli* positive control (DNA extracted from 10^2^ Macias strain epimastigotes)

Figure 3 shows a representative gel of the results generated by the multiplex conventional PCR for the screening of *T. cruzi* natural infection in field-collected triatomines. The presence of the 163 bp fragment corresponding to the triatomine 12S rRNA gene, indicated no PCR inhibition in the analyzed insect samples. The absence of the 330 bp kDNA fragment (Lane 9) confirmed the sample as a “true negative” result for *T. cruzi* DNA considering the amplification of the internal control triatomine gene. In addition, *T. rangeli* DNA was not detected in field-collected triatomines, since no amplification of both kDNA fragments (360 and 760 bp) was observed in all analyzed samples.

**Fig. 4:**
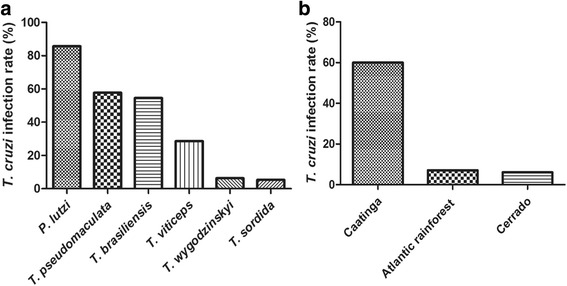
*Trypanosoma cruzi* detection among distinct triatomine species and biomes from Brazil. *T. cruzi* DNA detection was performed by multiplex cPCR, and the results were compared between distinct field-collected triatomine species (**a**) and the geographical regions or biomes where insects were captured (**b**)

When PCR-positive samples were analyzed by triatomine species (Fig. 4a), the highest infection rate was observed for *P. lutzi* (6/7, 85.7%), followed by *T. pseudomaculata* (15/26, 57.7%), *T. brasiliensis* (12/22, 54.5%), *T*. *vitticeps* (2/7, 28.6%), *T*. *wygodzinskyi* (3/48, 6.3%) and *T. sordida* (5/95, 5.3%). Caatinga biome yields the highest infection rate (60%) with 33 positive insects out of the 55 captured specimens. The positive kDNA-PCR samples corresponded to *T. pseudomaculata* (*n* = 15), *T. brasiliensis* (*n* = 12) and *P. lutzi* (*n* = 6). Concerning the Atlantic Rainforest and Cerrado biomes, the infection rates revealed by kDNA-PCR were lower, 7.1% (6 positive out of 84 collected insects) and 6.1% (4 positive out of 66 collected insects), respectively (Fig. 4b). The six positive specimens from the Atlantic Rainforest were *T. wygodzinskyi* (*n* = 3), *T. vitticeps* (*n* = 2) and *T. sordida* (*n* = 1), and four *T. sordida* samples were found positive in the Cerrado region.

Regarding microscopy observation, the three positive samples were related to *T*. *pseudomaculata*, *T. brasiliensis* and *P. lutzi* live adults, collected in Caatinga.

### *Trypanosoma cruzi* DNA quantification in triatomines

**Fig. 5:**
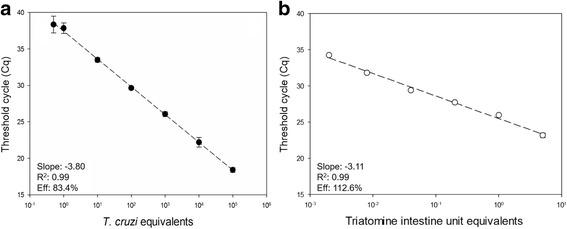
Reportable range for *T. cruzi* and triatomine intestine unit quantification by real-time qPCR. Multiplex *Taq*Man qPCR assays were carried out with serially diluted DNA extracted from reconstituted triatomine intestine samples containing *T. cruzi* epimastigotes, ranging from 10^5^ to 0.5 *T. cruzi* equivalents (**a**) and 5 to 0.002 triatomine intestine unit equivalents (**b**). The slope, R^2^ and amplification efficiency (Eff) are indicated in the chart

To quantify the parasite load in positive kDNA-PCR triatomine samples, a *Taq*Man-based multiplex qPCR was developed, targeting *T. cruzi* nuclear satellite DNA (SAT-DNA-qPCR) and the triatomine 12S rRNA gene. Following our methodology, detection of *T. cruzi* DNA was linear from 10^5^ to 0.5 parasite equivalents, using triatomine intestine samples (not infected) spiked with *T. cruzi* epimastigotes (Fig. 5a). Moreover, the detection of triatomine DNA was linear, ranging from 5 to 0.002 intestine unit equivalents (Fig. 5b). For the *T. cruzi* and triatomine targets in multiplex, PCR amplification efficiencies were 83.4 and 112.6% respectively, and the linearity coefficient (R^2^) was 0.99 for both targets, confirming the improved performance of the assay.

**Fig. 6:**
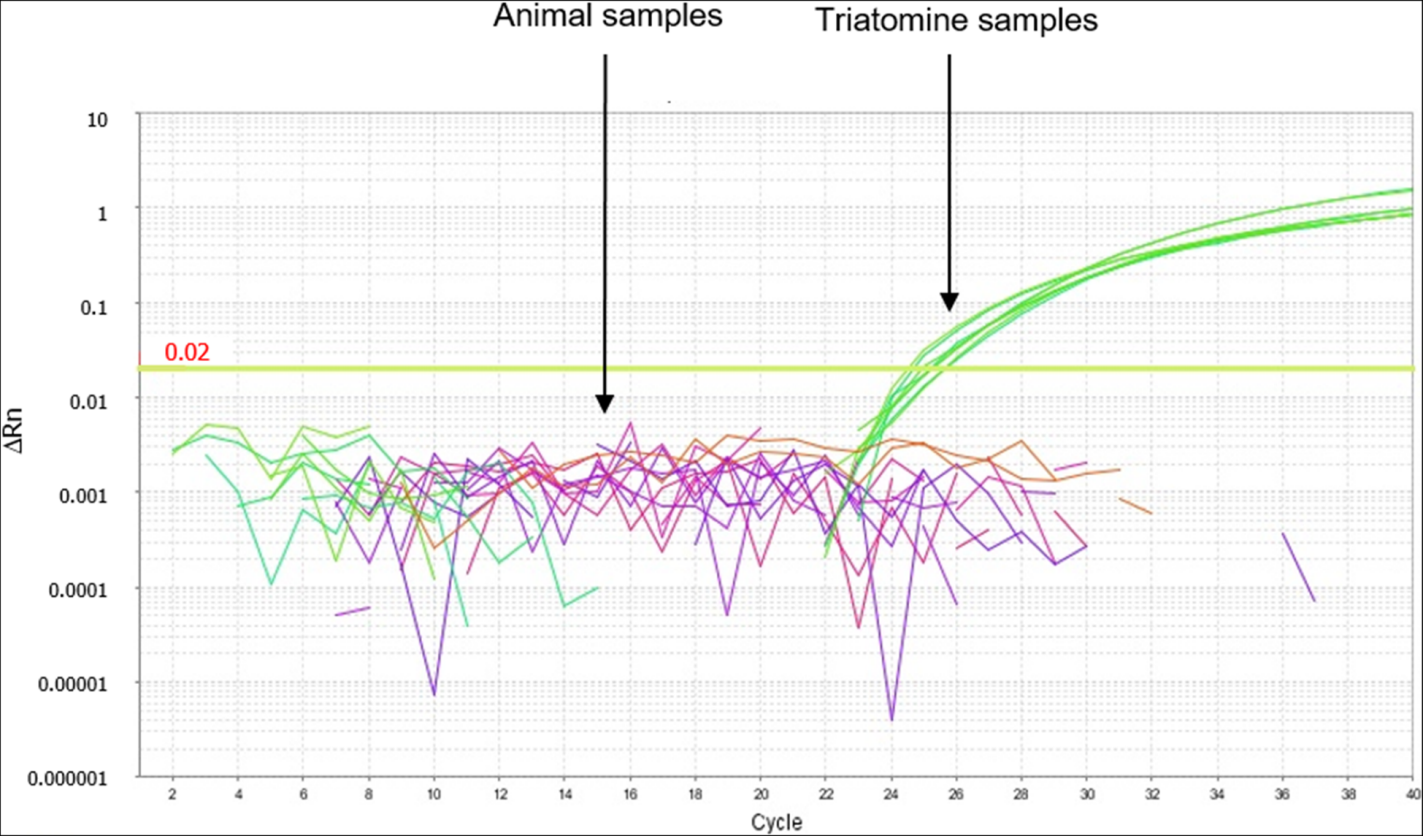
Detection of different triatomine species and interference of possible blood meal sources in the real time qPCR by analyzing the 12S rDNA gene. Representative amplification plots of DNA samples extracted from one intestine unit of different triatomine species (adults maintained in insectary) and absence of amplification for DNAs obtained from blood samples of possible meal sources for field-collected triatomines

The amplification signal for different triatomine species based on the 12S rDNA target, as well as the possible interference of blood-meal source in the multiplex real-time PCR assay, were investigated (Fig. 6, Table 3). There was no significant difference concerning the amplification of DNA extracted from one intestine of an adult insect (8–10 ng) from the species *R. prolixus, R. neglectus, T. vitticeps* and *T. sordida*, with C_q_ values varying from 24.54 ± 0.39 to 26.86 ± 1.37 (Kruskal-Wallis: *H* = 4.59, *df* = 3, *P* = 0.205). On the other hand, the interference of possible triatomine blood-meal sources was also investigated, and no amplification using DNA (20 ng) extracted from the blood of human, bat, cat, dog, opossum or rat was observed (Table 3).

**Table 3:** Representation of C_q_ mean values for detection of the 12S rDNA gene by real-time PCR in distinct triatomine species and absence of amplification signal in animal blood samples

Triatomine detection (1 intestine, 8–10 ng DNA)		Possible blood-meal source detection (blood, 20 ng DNA)	
Triatomine species	Cq (Mean ± SD)	Origin	Cq (Mean ± SD)
*Rhodnius prolixus*	25.54 ± 0.30	Human	nd
*Rhodnius neglectus*	26.55 ± 0.54	Bat	nd
*Triatoma vitticeps*	26.86 ± 1.37	Cat	nd
*Triatoma sordida*	24.54 ± 0.39	Dog	nd
		Opossum	nd
		Rat	nd

*Abbreviations*: *C*_*q*_ threshold cycle, *SD* standard deviation, *nd* not detected

Table 4 presents the results of SAT-DNA qPCR positivity to estimate LOD for *T. cruzi* DNA in triatomine reconstituted samples. A LOD of 0.41 parasite equivalents was found and the threshold cycle (C_q_) corresponding to the detection of one solely triatomine intestine, used as an internal reference, varied from 21.19 ± 0.39 to 23.06 ± 1.51 with the threshold set at 0.02.

**Table 4:** SAT-DNA qPCR positivity to estimate the limit of detection (LOD) for *T. cruzi* DNA in triatomine samples

Parasite load (*T. cruzi* equivalents)	Assay #	Positive samples (%)	Triatomine internal reference (C_q_ ± SD)
20	1	12 (100)	21.19 ± 0.39
	2	12 (100)	
	3	12 (100)	
4	1	12 (100)	22.09 ± 1.85
	2	12 (100)	
	3	12 (100)	
0.8	1	12 (100)	21.48 ± 0.86
	2	12 (100)	
	3	12 (100)	
0.16	1	10 (83.3)	22.56 ± 1.40
	2	8 (66.7)	
	3	10 (83.3)	
0.032	1	11 (91.7)	22.65 ± 1.12
	2	10 (83.3)	
	3	5 (41.7)	
0.0064	1	7 (58.3)	23.06 ± 1.51
	2	6 (50.0)	
	3	6 (50.0)	
0.00128	1	1 (8.3)	22.39 ± 0.76
	2	1 (8.3)	
	3	–	

*Abbreviations*: *C*_*q*_ threshold cycle, *SD* standard deviation

**Fig. 7:**
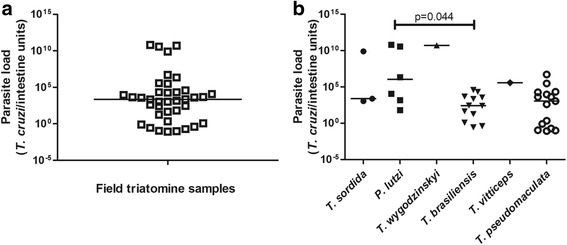
Distribution of parasite load in field triatomine samples. **a** *Trypanosoma cruzi* load estimated by multiplex real-time qPCR targeting the parasite nuclear satellite sequences (SAT-DNA-qPCR) and normalized by triatomine intestine units. The straight horizontal line indicates the median value. **b** Parasite loads and median values (horizontal lines) are indicated per triatomine species

The multiplex qPCR assay was used to estimate *T. cruzi* load in 38 out of the 43 field-collected triatomines that gave positive results by the kDNA-PCR test. A wide range parasite load distribution varying from 8.05 × 10^-2^ to 6.31 × 10^10^ was observed, with a median of 2.29 × 10^3^ (2.43 × 10^1^–2.10 × 10^4^) *T. cruzi*/intestine units (Fig. 7a). When parasite load was analyzed by triatomine species (Fig. 7b), a significantly greater median value was found for *P. lutzi* in comparison with *T. brasiliensis* (Shapiro-Wilk: *P <* 0.050, Mann-Whitney Rank Sum Test: *U* = 14, *T*_(6,12)_ = 79, *P* = 0.044). It was possible to infer the parasite load for only one specimen of *T*. *wygodzinskyi* and *T*. *vitticeps* each and for 3 out of 5 positive *T. sordida*.

## Discussion

Molecular diagnostic tests have been improved in the last years, with the development of conventional and quantitative real-time PCR-based assays for detection and quantification of *T. cruzi* DNA in different biological specimens. These include human blood and serum [26–28], animal tissues [29] and feces or digestive tract of triatomine vectors [30, 31]. The establishment of precise methodologies able to detect and quantify *T. cruzi* load in field-collected triatomine samples, would be important for monitoring natural infection rates in Chagas disease vectors and its further application for molecular epidemiological studies. Here, we report the development and application of PCR and *Taq*Man real-time qPCR assays, both in multiplex, for the detection and quantification of *T. cruzi* load in field-collected triatomines.

PCR-based bug infection rates may be underestimated due to PCR inhibitors. For this, DNA extraction methods from feces or digestive tract need to be improved in order to increase PCR sensitivity. In this study, triatomine guts were pretreated in a lysis solution containing proteinase K for 2 h at 56 °C, before DNA extraction using a modified protocol based on silica columns. The absence of PCR inhibition was demonstrated by amplification of the internal control reference (triatomine 12S rRNA gene) in both conventional and real-time PCR assays. From the latter, it was possible to observe slight variation on C_q_ values, from 21.19 ± 0.39 to 23.06 ± 1.51, for samples corresponding to one adult triatomine intestine unit containing 20 or 0.0064 *T. cruzi* equivalents, respectively (Table 4).

The kDNA-based PCR screening of field-collected triatomines was shown to be more sensitive than microscopic examination (ME) with a positivity of 21%, whereas only 7% of positive PCR results were confirmed by ME of fecal drops of live insects. In addition, all negative PCR samples were microscopically negative for *T. cruzi* infection. It should be noticed that the three triatomine specimens for which ME was able to identify *T. cruzi* flagellates in the feces (3/205, 1.5%) represented live adult insects, whereas the molecular analysis was performed with DNA obtained from midgut and rectum of live and dead insects. These technical features could in part explain the differences in positivity between microscopy and PCR, considering the lower number of parasites in triatomine-diluted feces when compared with intestinal content homogenates used for DNA extraction, and thus yielding false negative results by the traditional ME diagnosis.

A study using kDNA-PCR tests for screening of *T. cruzi* infection from fecal samples of field-collected triatomines in Argentina allowed the identification of 7.5% positive samples among those microscopically negative [32]. For few samples in which the presence of flagellates was evidenced by ME, both kDNA and nuclear satellite sequence of *T. cruzi* were not amplified. However, positive amplification of a 24Sα rDNA conserved fragment revealed infection with another trypanosomatid, suggesting that caution should be taken when diagnosing *T. cruzi* infection based only on ME of unstained fresh preparations of triatomine feces [32]. Similar evidence was described in a recent study in Brazil, in which, 46% of sylvatic *T. brasiliensis* collected in Rio Grande do Norte State were infected with *T. cruzi*-like parasites by ME of fecal drops, while using mini-exon PCR amplification, a lower infection prevalence was detected in the triatomine digestive tracts [33]. The authors suggested this was a result of misidentification of *T. cruzi*-like parasites as *T. cruzi* during direct ME thus generating false positive results for local Chagas disease control surveillance campaigns. Additionally, results obtained from first- and second-stage nymphs using ME are underestimated due to the difficulty in obtaining feces during these stages. This limitation is not observed when PCR is performed [15].

Differences in *T. cruzi* infection prevalence are observed between triatomine species [33, 34]. Regarding *T. brasiliensis*, the influence of feeding sources on the rate of *T. cruzi* infection in specimens collected in distinct sites and ecotopes in Rio Grande do Norte State has been demonstrated [33]. *Triatoma brasiliensis* is the most important vector species in northeastern Brazil, due to its wide geographical distribution, its rates of natural infection and capacity to inhabit both natural and human environments [35]. *Triatoma pseudomaculata* is the second epidemiologically more important species after *T. brasiliensis* in the northeast of Brazil [36]. Both are synanthropic species, endemic in the State of Ceará (Caatinga biome) and are easily found in municipalities where Chagas disease is reported [34, 37]. *Triatoma pseudomaculata* is more frequently found in peridomiciliary habitats and generally feeding on birds. Nevertheless, the domiciliary foci of this species have been observed in Ceará and Minas Gerais [38, 39]. Caatinga biome occupies the third position in triatomines diversity [40]. Subsistence agriculture is the main activity of the inhabitants, beyond cattle raising, mainly goats and the presence of chickens living free in peridomicile attracting blood-sucking insect vectors around human habitations [41]. In the present study, *T. pseudomaculata* and *T. brasiliensis* captured in the Caatinga biome, the State of Ceará, were the most frequent species captured in the region, with prevalence of 47.4 and 40%, respectively, and *T. cruzi* infection rates of 57.7 and 54.5%, respectively. The previous report in the municipality of Russas (Ceará) showed high rates of natural infection by *T. cruzi* in *T. brasiliensis* (90%) and *T*. *pseudomaculata* (80%) using PCR directed to the mini-exon region; although the authors considered these much higher than expected [42]. *Triatoma brasiliensis* is predominantly found in domestic environments of the semiarid region of Brazil [36]. Here, it is important to note that four out of 12 *T. brasiliensis-*positive specimens were captured in the intradomicile environment (one of these was also positive by microscopy), and the others were from the peridomicile environment. *Triatoma brasiliensis* was also found positive for *T. cruzi* in the domestic, peridomestic and wild environments in Tauá (Ceará State), with the highest infection rate of 14% in the peridomicile [43].

In our study, of the few specimens (*n* = 7) of *P. lutzi* captured in Caatinga, in the peridomicile environment, only one was negative for *T. cruzi* kDNA thus yielding the highest infection prevalence for this species when compared with all others (85.7%) (Fig. 4). Of the six positive adult specimens, five were found at the same site of collection (Additional file 1: Table S1). *Panstrongylus lutzi* is one of the native species of northeastern Brazil, and its geographical distribution coincides with the extension of the Caatinga biome. Mainly in Ceará and Pernambuco states, it is increasingly common the presence of adults in the intradomicile and generally presenting high rates of *T. cruzi* infection, elevating the epidemiological importance of this species [44]. Studies evaluating natural infections by *T. cruzi* in *P. lutzi* have been reported using parasitological search by microscopy; in one of these, *T. cruzi* flagellates were identified in 29.1% of the specimens found in the intradomicile, in 20 municipalities of Ceará [44]. As mentioned, the high positivity found in the present study for kDNA-PCR may have been overestimated due to the collection of a small number of *P. lutzi* specimens and the vast majority being infected.

Although the number of collected bugs in the Brazilian Atlantic Rainforest and Cerrado biomes was higher than in Caatinga, *T. cruzi* infection rates were lower (7.1 and 6.1%, respectively). Nowadays, in Brazil, *T. sordida* merits special attention due to its wide distribution (Atlantic Rainforest, Caatinga, Cerrado and Amazon biomes), tendency to invade domestic environments and vectorial competence [45]. This species is endemic in Cerrado, the main biome of Central Brazil, more frequently found in the peridomestic environment, especially in chicken coops, occupying areas where *T. infestans* has been eliminated. *Triatoma sordida* is usually considered as a secondary vector of *T. cruzi* and usually exhibits low rates of natural infection [46, 47]; this species is associated with the reinfestation of dwellings treated with insecticides [48], and is the most common synanthropic species captured in the Central-West region of Brazil [49]. These features are in agreement with our findings, where *T. sordida* was the main species captured in both, Cerrado and Atlantic Forest biomes, with frequencies of 62.1 and 64.3%, respectively. In the Cerrado, four specimens out of 41 yielded positive for *T. cruzi* kDNA (9.8% infection rate). In another investigation, 10.7% of *T. sordida* specimens captured in Mato Grosso do Sul were positive for flagellated protozoa, as determined by microscopic search, and 18.1% were positive for *T. cruzi* using kDNA-PCR [50]. The latter value was almost two-fold higher than that observed herein (9.8%), for specimens collected in different areas of the Cerrado biome, including the State of Mato Grosso do Sul, and none were found positive by direct microscopy. In the Atlantic Rainforest, despite the high prevalence of *T. sordida* captured in the peridomicile (64.3%), only one specimen out of 54 was positive for *T. cruzi* kDNA (1.9%). Overall, comparing the triatomine species evaluated herein, *T. sordida* revealed the lowest prevalence of *T. cruzi* infection (5.3%, Fig. 4a).

*Triatoma wygodzinskyi* is a rupicolous species, restricted to the sylvatic environment and has received scarce attention due to its limited epidemiological importance as a vector of *T. cruzi* [51]. This triatomine was initially described among a small number of specimens collected in southern Minas Gerais and São Paulo states [21]. Environmental information provided by remote sensors predicted a more extensive geographic distribution for *T*. *wygodzinskyi*, including the states of Rio de Janeiro, Paraná, Santa Catarina and Rio Grande do Sul [52]. In our study, this species was captured in sylvatic environments of Cerrado and Atlantic Rainforest biomes, with a prevalence of 37.9 and 27.4%, respectively, and presented three positive specimens for *T. cruzi* kDNA that were only found in the Atlantic forest (6.3% infection rate).

*Triatoma vitticeps* has a geographical distribution more restricted to the Atlantic Rainforest [40], with adult specimens being captured in rural areas in Rio de Janeiro, Bahia, Minas Gerais and Espírito Santo [53, 54]. The species colonizes peridomiciliary areas, increasing the risk of *T. cruzi* transmission to humans [54]. In a recent survey conducted in the northeastern region of Minas Gerais, *T*. *vitticeps* was present throughout almost all the municipalities, predominantly in its adult form, in the intradomicile [55]. In the present work, *T*. *vitticeps* corresponded to 8.3% of the specimens collected in the Atlantic Rainforest biome, in the peridomicile environments of two municipalities of Rio de Janeiro, and revealed an infection rate of 28.6% (two positives out of seven collected). In 1998, an investigation was conducted in the municipal district of Santa Maria Madalena, Rio de Janeiro State, where *T. cruzi* prevalence in *T*. *vitticeps* adult triatomines in the intradomicile was estimated as 79% [56]. The higher infection rate of 86.2% by flagellates similar to *T. cruzi* was found in the Espírito Santo State for sylvatic *T*. *vitticeps*; 47.4% of 116 analyzed insects were captured in the bedroom of residences of which 85% were infected [53]. In spite of the occasional invasion of houses by *T*. *vitticeps* in Rio de Janeiro State [56–59] and its recognized role in the vectorial transmission of Chagas disease in Espírito Santo State [60–62], the extended interval between feeding and defecation reduces the success for the classical vectorial route transmission and could in part contribute to the low incidence of Chagas disease in both states [62, 63].

To the best of our knowledge, to date there is no study comparing *T. cruzi* parasite load among field-collected triatomine species or per geographical area. Here, 38 of the 43 triatomines with positive results for *T. cruzi* kDNA were quantified by the developed *Taq*Man multiplex qPCR. Not all kDNA-PCR positive samples could be confirmed by SAT-DNA-qPCR, which is expected from the fewer copies of satellite DNA sequences in the *T. cruzi* DTU - TcI [64]. Although molecular characterization of *T. cruzi* populations was not performed in this study, it is known that TcI displays the first largest distribution in both, wild or domestic transmission cycles [65], beyond being very frequently found in concomitant infection with other DTUs in triatomine bugs and seemed to be the predominant isolate, even in mixed infections [66]. A higher sensitivity of kDNA compared to SAT-DNA-qPCR to evaluate human parasitaemia in Chagas disease patients has also been confirmed [27].

To estimate parasite load in field-collected triatomines, a highly sensitive *Taq*Man multiplex qPCR was developed with a LOD of 0.41 *T. cruzi*/intestine units. This is in agreement with the one reported for other tissues and organisms such as human blood, also using *T. cruzi* nuclear satellite DNA as a target [67]. In addition, the assay enabled amplification of DNA from intestine samples of different triatomine species with similar C_q_ values, indicating a low variance in the 12S rDNA copy number in triatomine genomes, which contributes to the reproducibility of the assay. Also, possible blood-meal sources of field-collected triatomines, such as human, bat, cat, dog, opossum or rat did not interfere in the multiplex real-time PCR assay, showing the improved specificity of this methodology.

The observed dynamic extension provided linear quantification in at least 6-log range, for both *T. cruzi* and triatomines, allowing an accurate normalization of parasite load. This normalization is essential to compensate variations between samples, such as DNA amount, that depends on the developmental stage of the insect and the time post-feeding, beyond the quality of sample preservation and differences in size between species. Following our methodology, it is possible to perform a direct comparison between field samples from different species and geographical localities.

In spite of the wide range of parasite load values observed in the present study among field-collected triatomines (*c*.10^-2^–10^10^ *T. cruzi*/intestine units), a significant difference in the median values was only observed between *P. lutzi* and *T. brasiliensis* (*P* = 0.044, Fig. 7b). In two *P. lutzi* specimens, the number of *T. cruzi*/intestine units exceeded 10^10^, as also observed for one specimen of *T. sordida* and for the single *T*. *wygodzinskyi* sample; whereas for *T. brasiliensis*, the highest parasite load did not exceed 10^5^ *T. cruzi*/intestine units (Fig. 7b). Studies on trypanosome-triatomine interactions may allow the identification of humoral and cellular mechanisms in the vectors that either facilitate or prevent the transmission of parasites in different vector species, acting as limiting factors for the development of the parasite [68–70]. The susceptibility of a vector depends on various factors, including digestive enzymes, hemolysins, agglutinins, microbiota and especially antimicrobial factors, which are potentially involved in regulating the development of *T. cruzi* in the gut [70]. Moreover, differential regulation of parasite populations between different strains of *T. cruzi* has also been demonstrated [70]. In spite of not presenting data of *T. cruzi* genotyping, our findings reproduce the magnitude of parasitism found in field-collected triatomines. This novel quantitative real-time PCR raises the possibility for further evaluating parasite load, as a promising biomarker of vectorial capacity, through investigating the correlation between the amount of *T. cruzi* found in different portions of the insect midgut and the triatomine ability to transfer the parasite during a blood-meal.

## Conclusions

Our results present a novel multiplex PCR-based approach, as a combination of conventional and real-time PCR methodologies, to monitor *T. cruzi* infection in triatomines. Both methods can be successfully applied to epidemiological studies of Chagas disease surveillance, and for evaluating *T. cruzi* life-cycle and its colonization in the digestive tract of triatomine experimental models. Parasite load quantification in triatomines emerges as a potential biomarker to evaluate vectorial capacity.

## Additional file

Additional file 1: Table S1. Detailed description of field-collected triatomine specimens from 26 municipalities of three Brazilian biomes. (DOC 350 kb)

## Abbreviations

BHI: Brain heart infusion
ChD: Chagas disease
COLPROT: Coleção de Protozoários da Fundação Oswaldo Cruz, Rio de Janeiro, Brazil
cPCR: Conventional PCR
C_q_: Threshold cycle
DTU: Discrete typing unit
Fiocruz: Oswaldo Cruz Foundation
kDNA: Kinetoplast deoxyribonucleic acid
LOD: Limit of detection
NFQ-MGB: Non fluorescent quencher-minor groove binding
qPCR: Quantitative real-time PCR
R^2^: Coefficient of linearity
SAT-DNA: Satellite deoxyribonucleic acid
TcI, TcIV and TcVI: *Trypanosoma cruzi* discrete typing units I, IV and VI
UFF: Fluminense Federal University
