# Supplementary material for: Development of conventional and real-time multiplex PCR-based assays for estimation of natural infection rates and Trypanosoma cruzi load in triatomine vectors
Source: Parasit Vectors. 2017 Aug 29;10:404. doi: 10.1186/s13071-017-2343-x (PMC5576278; doi:10.1186/s13071-017-2343-x)
Supplement: Additional file 1: Table S1. — Detailed description of field-collected triatomine specimens from 26 municipalities of three Brazilian biomes. (DOC 350 kb) [file 13071_2017_2343_MOESM1_ESM.doc]

**Additional file 1: Table S1.** Detailed description of field triatomine specimens collected in 26 municipalities of three Brazilian biomes.

| **Biome** | **State** | **Locality** | **Species** | **Life stage** | **Environment** | **Conventional PCR** | **Microscopy** | | **Parasite load (Mean)** |
| --- | --- | --- | --- | --- | --- | --- | --- | --- | --- |
| **Caatinga** | Ceará | Santa Quitéria | *Triatoma brasiliensis* | N4 | Peridomicile A | **POS** | NEG | | 1.80E+01 |
|  | Ceará | Santa Quitéria | *Triatoma brasiliensis* | N5 | Peridomicile A | **POS** | NEG | | 2.8E-01 |
|  | Ceará | Santa Quitéria | *Triatoma brasiliensis* | Female | Peridomicile A | NEG | NEG | | - |
|  | Ceará | Santa Quitéria | *Triatoma brasiliensis* | N3 | Peridomicile A | NEG | NEG | | - |
|  | Ceará | Santa Quitéria | *Triatoma brasiliensis* | N4 | Peridomicile A | NEG | NEG | | - |
|  | Ceará | Santa Quitéria | *Triatoma brasiliensis* | N5 | Peridomicile A | NEG | NEG | | - |
|  | Ceará | Santa Quitéria | *Triatoma brasiliensis* | N4 | Peridomicile A | NEG | NEG | | - |
|  | Ceará | Santa Quitéria | *Triatoma brasiliensis* | N5 | Peridomicile A | NEG | NEG | | - |
|  | Ceará | Santa Quitéria | *Triatoma brasiliensis* | Female | Peridomicile A | NEG | NEG | | - |
|  | Ceará | Farias Brito | *Triatoma brasiliensis* | Male | Peridomicile A | **POS** | NEG | | 3.73E+04 |
|  | Ceará | Farias Brito | *Triatoma brasiliensis* | Male | Peridomicile A | **POS** | NEG | | 2.19E+03 |
|  | Ceará | Assaré | *Triatoma brasiliensis* | Male | Intradomicile A | **POS** | **POS** | | 4.30E+01 |
|  | Ceará | Assaré | *Triatoma brasiliensis* | Male | Peridomicile A | NEG | NEG | | - |
|  | Ceará | Campos Sales | *Triatoma brasiliensis* | N5 | Peridomicile A | **POS** | NEG | | 4.70E+03 |
|  | Ceará | Campos Sales | *Triatoma brasiliensis* | N5 | Peridomicile A | **POS** | NEG | | 2.10E+04 |
|  | Ceará | Campos Sales | *Triatoma brasiliensis* | N5 | Peridomicile A | **POS** | NEG | | 2.60E+02 |
|  | Ceará | Campos Sales | *Triatoma brasiliensis* | Female | Intradomicile A | **POS** | NEG | | 2.80E+02 |
|  | Ceará | Campos Sales | *Triatoma brasiliensis* | Female | Intradomicile A | **POS** | NEG | | 1.20E+00 |
|  | Ceará | Campos Sales | *Triatoma brasiliensis* | Female | Intradomicile A | **POS** | NEG | | 4.00E-01 |
|  | Ceará | Potengi | *Triatoma brasiliensis* | N5 | Peridomicile A | **POS** | NEG | | 3.60E+03 |
|  | Ceará | Potengi | *Triatoma brasiliensis* | N5 | Peridomicile A | NEG | NEG | | - |
|  | Ceará | Potengi | *Triatoma brasiliensis* | N5 | Peridomicile A | NEG | NEG | | - |
|  | Ceará | Santa Quitéria | *Panstrongylus lutzi* | Female | Peridomicile A | **POS** | **POS** | | 6.66E+01 |
|  | Ceará | Santa Quitéria | *Panstrongylus lutzi* | Male | Peridomicile A | **POS** | NEG | | 3.74E+10 |
|  | Ceará | Santa Quitéria | *Panstrongylus lutzi* | Male | Peridomicile A | **POS** | NEG | | 6.31E+10 |
|  | Ceará | Santa Quitéria | *Panstrongylus lutzi* | Male | Peridomicile A | **POS** | NEG | | 2.20E+06 |
|  | Ceará | Santa Quitéria | *Panstrongylus lutzi* | Female | Peridomicile A | NEG | NEG | | - |
|  | Ceará | Santa Quitéria | *Panstrongylus lutzi* | Male | Peridomicile A | **POS** | NEG | | 1.25E+04 |
|  | Ceará | Nova Olinda | *Panstrongylus lutzi* | Male | Peridomicile A | **POS** | NEG | | 1.50E+03 |
|  | Ceará | Santa Quitéria | *Triatoma pseudomaculata* | Male | Peridomicile A | **POS** | **POS** | | 1.14E+03 |
|  | Ceará | Araripe | *Triatoma pseudomaculata* | Female | Peridomicile A | **POS** | NEG | | 5.11E+06 |
|  | Ceará | Araripe | *Triatoma pseudomaculata* | Female | Peridomicile B | **POS** | NEG | | 9.80E-02 |
|  | Ceará | Araripe | *Triatoma pseudomaculata* | Male | Peridomicile C | **POS** | NEG | | 1.10E-01 |
|  | Ceará | Araripe | *Triatoma pseudomaculata* | Female | Peridomicile C | **POS** | NEG | | 1.90E-01 |
|  | Ceará | Araripe | *Triatoma pseudomaculata* | N5 | Peridomicile A | NEG | NEG | | - |
|  | Ceará | Araripe | *Triatoma pseudomaculata* | N5 | Peridomicile C | NEG | NEG | | - |
|  | Ceará | Farias Brito | *Triatoma pseudomaculata* | N5 | Peridomicile B | **POS** | NEG | | 4.20E+03 |
|  | Ceará | Farias Brito | *Triatoma pseudomaculata* | Female | Peridomicile B | NEG | NEG | | - |
|  | Ceará | Farias Brito | *Triatoma pseudomaculata* | Female | Peridomicile B | NEG | NEG | | - |
|  | Ceará | Farias Brito | *Triatoma pseudomaculata* | N5 | Peridomicile B | NEG | NEG | | - |
|  | Ceará | Farias Brito | *Triatoma pseudomaculata* | N5 | Peridomicile B | **POS** | NEG | | 8.50E-02 |
|  | Ceará | Potengi | *Triatoma pseudomaculata* | N5 | Peridomicile A | **POS** | NEG | | 8.00E-01 |
|  | Ceará | Potengi | *Triatoma pseudomaculata* | N5 | Peridomicile A | **POS** | NEG | | 2.20E+00 |
|  | Ceará | Potengi | *Triatoma pseudomaculata* | N5 | Peridomicile A | **POS** | NEG | | 4.00E+02 |
|  | Ceará | Potengi | *Triatoma pseudomaculata* | Female | Peridomicile B | **POS** | NEG | | 3.02E+05 |
|  | Ceará | Potengi | *Triatoma pseudomaculata* | Female | Peridomicile B | **POS** | NEG | | 1.80E+04 |
|  | Ceará | Potengi | *Triatoma pseudomaculata* | Female | Peridomicile B | **POS** | NEG | | 9.10E+03 |
|  | Ceará | Potengi | *Triatoma pseudomaculata* | N5 | Peridomicile C | **POS** | NEG | | 2.10E+04 |
|  | Ceará | Potengi | *Triatoma pseudomaculata* | N5 | Peridomicile C | **POS** | NEG | | 4.00E+03 |
|  | Ceará | Potengi | *Triatoma pseudomaculata* | N5 | Peridomicile C | NEG | NEG | | - |
|  | Ceará | Potengi | *Triatoma pseudomaculata* | N5 | Peridomicile C | NEG | NEG | | - |
|  | Ceará | Potengi | *Triatoma pseudomaculata* | Male | Peridomicile C | NEG | NEG | | - |
|  | Ceará | Potengi | *Triatoma pseudomaculata* | N5 | Peridomicile B | NEG | NEG | | - |
|  | Ceará | Potengi | *Triatoma pseudomaculata* | N5 | Peridomicile B | NEG | NEG | | - |
|  | Ceará | Potengi | *Triatoma pseudomaculata* | Male | Peridomicile B | NEG | NEG | | - |
| **Cerrado** | Tocantins | Aurora do Tocantins | *Triatoma sordida* | Female | Peridomicile A | **POS** | NEG | | 1.12E+03 |
|  | Tocantins | Aurora do Tocantins | *Triatoma sordida* | Male | Peridomicile A | NEG | NEG | | - |
|  | Tocantins | Aurora do Tocantins | *Triatoma sordida* | Male | Peridomicile A | NEG | NEG | | - |
|  | Tocantins | Aurora do Tocantins | *Triatoma sordida* | Male | Peridomicile A | NEG | NEG | | - |
|  | Tocantins | Aurora do Tocantins | *Triatoma sordida* | Female | Peridomicile A | NEG | NEG | | - |
|  | Tocantins | Aurora do Tocantins | *Triatoma sordida* | Female | Peridomicile A | NEG | NEG | | - |
|  | Tocantins | Combinado | *Triatoma sordida* | Male | Peridomicile A | NEG | NEG | | - |
|  | Tocantins | Combinado | *Triatoma sordida* | Female | Peridomicile A | NEG | NEG | | - |
|  | Tocantins | Combinado | *Triatoma sordida* | Male | Peridomicile A | NEG | NEG | | - |
|  | Tocantins | Combinado | *Triatoma sordida* | Female | Peridomicile A | NEG | NEG | | - |
|  | Bahia | Lavandeira | *Triatoma sordida* | Male | Peridomicile A | NEG | NEG | | - |
|  | Bahia | São Desidério | *Triatoma sordida* | Female | Peridomicile A | NEG | NEG | | - |
|  | Bahia | São Desidério | *Triatoma sordida* | Male | Peridomicile A | NEG | NEG | | - |
|  | Bahia | São Desidério | *Triatoma sordida* | Male | Peridomicile A | NEG | NEG | | - |
|  | Bahia | São Desidério | *Triatoma sordida* | Male | Peridomicile A | NEG | NEG | | - |
|  | Bahia | São Desidério | *Triatoma sordida* | Female | Peridomicile A | NEG | NEG | | - |
|  | Mato Grosso do Sul | Terenos | *Triatoma sordida* | Male | Peridomicile A | **POS** | NEG | | 2.37E+03 |
|  | Mato Grosso do Sul | Terenos | *Triatoma sordida* | Female | Peridomicile A | NEG | NEG | | - |
|  | Mato Grosso do Sul | Terenos | *Triatoma sordida* | Female | Peridomicile A | NEG | NEG | | - |
|  | Mato Grosso do Sul | Terenos | *Triatoma sordida* | Female | Peridomicile A | NEG | NEG | | - |
|  | Mato Grosso do Sul | Terenos | *Triatoma sordida* | Female | Peridomicile A | NEG | NEG | | - |
|  | Minas Gerais | Bocaiúva | *Triatoma sordida* | Female | Peridomicile A | NEG | NEG | | - |
|  | Minas Gerais | Bocaiúva | *Triatoma sordida* | Male | Peridomicile B | NEG | NEG | | - |
|  | Minas Gerais | Bocaiúva | *Triatoma sordida* | Male | Peridomicile A | NEG | NEG | | - |
|  | Minas Gerais | Bocaiúva | *Triatoma sordida* | Male | Peridomicile A | NEG | NEG | | - |
|  | Minas Gerais | Bocaiúva | *Triatoma sordida* | Male | Peridomicile B | NEG | NEG | | - |
|  | Minas Gerais | Bocaiúva | *Triatoma sordida* | Male | Peridomicile B | NEG | NEG | | - |
|  | Minas Gerais | Bocaiúva | *Triatoma sordida* | Male | Peridomicile A | NEG | NEG | | - |
|  | Minas Gerais | Guaraciama | *Triatoma sordida* | Male | Peridomicile A | NEG | NEG | | - |
|  | Minas Gerais | Guaraciama | *Triatoma sordida* | Male | Peridomicile A | NEG | NEG | | - |
|  | Minas Gerais | Guaraciama | *Triatoma sordida* | Female | Peridomicile A | NEG | NEG | | - |
|  | Minas Gerais | Guaraciama | *Triatoma sordida* | Male | Peridomicile A | NEG | NEG | | - |
|  | Minas Gerais | Guaraciama | *Triatoma sordida* | Male | Peridomicile A | NEG | NEG | | - |
|  | Minas Gerais | Itaobim | *Triatoma sordida* | Female | Peridomicile A | **POS** | NEG | | 7.94E+09 |
|  | Minas Gerais | Itaobim | *Triatoma sordida* | Male | Peridomicile A | NEG | NEG | | - |
|  | Minas Gerais | Itaobim | *Triatoma sordida* | Female | Peridomicile A | NEG | NEG | | - |
|  | Minas Gerais | Itaobim | *Triatoma sordida* | Male | Peridomicile A | NEG | NEG | | - |
|  | Minas Gerais | Itaobim | *Triatoma sordida* | Male | Peridomicile A | NEG | NEG | | - |
|  | Minas Gerais | Itaobim | *Triatoma sordida* | Male | Peridomicile A | NEG | NEG | | - |
|  | Minas Gerais | Itaobim | *Triatoma sordida* | Male | Peridomicile A | NEG | NEG | | - |
|  | Minas Gerais | Itaobim | *Triatoma sordida* | Male | Peridomicile A | NEG | NEG | | - |
|  | São Paulo | Sertãozinho | *Triatoma wygodzinskyi* | N2 | Sylvatic A | **POS** | - | | 5.17E+10 |
|  | São Paulo | Sertãozinho | *Triatoma wygodzinskyi* | N2 | Sylvatic A | **POS** | - | | Not detected |
|  | São Paulo | Sertãozinho | *Triatoma wygodzinskyi* | N2 | Sylvatic A | **POS** | - | | Not detected |
|  | São Paulo | Sertãozinho | *Triatoma wygodzinskyi* | N2 | Sylvatic A | NEG | - | | - |
|  | São Paulo | Sertãozinho | *Triatoma wygodzinskyi* | N2 | Sylvatic A | NEG | - | | - |
|  | São Paulo | Sertãozinho | *Triatoma wygodzinskyi* | N2 | Sylvatic A | NEG | - | | - |
|  | São Paulo | Sertãozinho | *Triatoma wygodzinskyi* | N2 | Sylvatic A | NEG | - | | - |
|  | São Paulo | Sertãozinho | *Triatoma wygodzinskyi* | N2 | Sylvatic A | NEG | - | | - |
|  | São Paulo | Sertãozinho | *Triatoma wygodzinskyi* | N2 | Sylvatic A | NEG | - | | - |
|  | São Paulo | Sertãozinho | *Triatoma wygodzinskyi* | N2 | Sylvatic A | NEG | - | | - |
|  | São Paulo | Sertãozinho | *Triatoma wygodzinskyi* | N2 | Sylvatic A | NEG | - | | - |
|  | São Paulo | Sertãozinho | *Triatoma wygodzinskyi* | N2 | Sylvatic A | NEG | - | | - |
|  | São Paulo | Sertãozinho | *Triatoma wygodzinskyi* | N2 | Sylvatic A | NEG | - | | - |
|  | São Paulo | Sertãozinho | *Triatoma wygodzinskyi* | N1 | Sylvatic A | NEG | - | | - |
|  | São Paulo | Sertãozinho | *Triatoma wygodzinskyi* | N1 | Sylvatic A | NEG | - | | - |
|  | São Paulo | Sertãozinho | *Triatoma wygodzinskyi* | N1 | Sylvatic A | NEG | - | | - |
|  | São Paulo | Sertãozinho | *Triatoma wygodzinskyi* | N1 | Sylvatic A | NEG | - | | - |
|  | São Paulo | Sertãozinho | *Triatoma wygodzinskyi* | N1 | Sylvatic A | NEG | - | | - |
|  | São Paulo | Sertãozinho | *Triatoma wygodzinskyi* | N1 | Sylvatic A | NEG | - | | - |
|  | São Paulo | Sertãozinho | *Triatoma wygodzinskyi* | N1 | Sylvatic A | NEG | - | | - |
|  | São Paulo | Sertãozinho | *Triatoma wygodzinskyi* | N1 | Sylvatic A | NEG | - | | - |
|  | São Paulo | Sertãozinho | *Triatoma wygodzinskyi* | N1 | Sylvatic A | NEG | - | | - |
|  | São Paulo | Sertãozinho | *Triatoma wygodzinskyi* | N1 | Sylvatic A | NEG | - | | - |
|  | São Paulo | Sertãozinho | *Triatoma wygodzinskyi* | Female | Sylvatic A | NEG | NEG | | - |
|  | São Paulo | Sertãozinho | *Triatoma wygodzinskyi* | N4 | Sylvatic A | NEG | NEG | | - |
| **Atlantic**  **Rainforest** | Mato Grosso do Sul | Douradina | *Triatoma sordida* | Male | Peridomicile A | NEG | NEG | | - |
|  | Mato Grosso do Sul | Douradina | *Triatoma sordida* | Female | Peridomicile A | NEG | NEG | | - |
|  | Mato Grosso do Sul | Douradina | *Triatoma sordida* | Male | Peridomicile A | NEG | NEG | | - |
|  | Mato Grosso do Sul | Douradina | *Triatoma sordida* | Male | Peridomicile A | NEG | NEG | | - |
|  | Mato Grosso do Sul | Aparecida do Taboado | *Triatoma sordida* | Male | Peridomicile A | **POS** | NEG | Not detected | |
|  | Mato Grosso do Sul | Aparecida do Taboado | *Triatoma sordida* | Female | Peridomicile A | NEG | NEG | - | |
|  | Mato Grosso do Sul | Aparecida do Taboado | *Triatoma sordida* | Female | Peridomicile A | NEG | NEG | - | |
|  | Mato Grosso do Sul | Aparecida do Taboado | *Triatoma sordida* | Male | Peridomicile A | NEG | NEG | - | |
|  | Mato Grosso do Sul | Aparecida do Taboado | *Triatoma sordida* | Female | Peridomicile A | NEG | NEG | - | |
|  | Mato Grosso do Sul | Aparecida do Taboado | *Triatoma sordida* | Female | Peridomicile A | NEG | NEG | - | |
|  | Mato Grosso do Sul | Aparecida do Taboado | *Triatoma sordida* | Male | Peridomicile A | NEG | NEG | - | |
|  | Mato Grosso do Sul | Aparecida do Taboado | *Triatoma sordida* | Male | Peridomicile A | NEG | NEG | - | |
|  | Mato Grosso do Sul | Aparecida do Taboado | *Triatoma sordida* | Male | Peridomicile A | NEG | NEG | - | |
|  | Mato Grosso do Sul | Aparecida do Taboado | *Triatoma sordida* | Female | Peridomicile A | NEG | NEG | - | |
|  | Mato Grosso do Sul | Aparecida do Taboado | *Triatoma sordida* | Female | Peridomicile A | NEG | NEG | - | |
|  | Mato Grosso do Sul | Aparecida do Taboado | *Triatoma sordida* | Male | Peridomicile A | NEG | NEG | - | |
|  | Mato Grosso do Sul | Aparecida do Taboado | *Triatoma sordida* | Female | Peridomicile A | NEG | NEG | - | |
|  | Mato Grosso do Sul | Três Lagoas | *Triatoma sordida* | Female | Peridomicile A | **POS** | NEG | Not detected | |
|  | Mato Grosso do Sul | Três Lagoas | *Triatoma sordida* | Male | Peridomicile A | NEG | NEG | - | |
|  | Mato Grosso do Sul | Três Lagoas | *Triatoma sordida* | Female | Peridomicile A | NEG | NEG | - | |
|  | Mato Grosso do Sul | Três Lagoas | *Triatoma sordida* | Female | Peridomicile A | NEG | NEG | - | |
|  | Mato Grosso do Sul | Três Lagoas | *Triatoma sordida* | Male | Peridomicile A | NEG | NEG | - | |
|  | Mato Grosso do Sul | Três Lagoas | *Triatoma sordida* | Female | Peridomicile A | NEG | NEG | - | |
|  | Mato Grosso do Sul | Três Lagoas | *Triatoma sordida* | Male | Peridomicile A | NEG | NEG | - | |
|  | Mato Grosso do Sul | Três Lagoas | *Triatoma sordida* | Male | Peridomicile A | NEG | NEG | - | |
|  | Mato Grosso do Sul | Três Lagoas | *Triatoma sordida* | Male | Peridomicile A | NEG | NEG | - | |
|  | Mato Grosso do Sul | Três Lagoas | *Triatoma sordida* | Female | Peridomicile A | NEG | NEG | - | |
|  | Mato Grosso do Sul | Três Lagoas | *Triatoma sordida* | Female | Peridomicile A | NEG | NEG | - | |
|  | Mato Grosso do Sul | Três Lagoas | *Triatoma sordida* | Male | Peridomicile A | NEG | NEG | - | |
|  | Mato Grosso do Sul | Três Lagoas | *Triatoma sordida* | Male | Peridomicile A | NEG | NEG | - | |
|  | Mato Grosso do Sul | Três Lagoas | *Triatoma sordida* | Female | Peridomicile A | NEG | NEG | - | |
|  | Mato Grosso do Sul | Três Lagoas | *Triatoma sordida* | Female | Peridomicile A | NEG | NEG | - | |
|  | Mato Grosso do Sul | Paranaíba | *Triatoma sordida* | Male | Peridomicile A | NEG | NEG | - | |
|  | Mato Grosso do Sul | Paranaíba | *Triatoma sordida* | Female | Peridomicile A | NEG | NEG | - | |
|  | Mato Grosso do Sul | Paranaíba | *Triatoma sordida* | Female | Peridomicile A | NEG | NEG | - | |
|  | Mato Grosso do Sul | Paranaíba | *Triatoma sordida* | Female | Peridomicile A | NEG | NEG | - | |
|  | Mato Grosso do Sul | Paranaíba | *Triatoma sordida* | Male | Peridomicile B | NEG | NEG | - | |
|  | Mato Grosso do Sul | Paranaíba | *Triatoma sordida* | Female | Peridomicile A | NEG | NEG | - | |
|  | Mato Grosso do Sul | Paranaíba | *Triatoma sordida* | Male | Peridomicile A | NEG | NEG | - | |
|  | Mato Grosso do Sul | Paranaíba | *Triatoma sordida* | Male | Peridomicile A | NEG | NEG | - | |
|  | Mato Grosso do Sul | Paranaíba | *Triatoma sordida* | Female | Peridomicile A | NEG | NEG | - | |
|  | Mato Grosso do Sul | Paranaíba | *Triatoma sordida* | Female | Peridomicile A | NEG | NEG | - | |
|  | Mato Grosso do Sul | Paranaíba | *Triatoma sordida* | Female | Peridomicile A | NEG | NEG | - | |
|  | Mato Grosso do Sul | Paranaíba | *Triatoma sordida* | Female | Peridomicile A | NEG | NEG | - | |
|  | Mato Grosso do Sul | Paranaíba | *Triatoma sordida* | Female | Peridomicile A | NEG | NEG | - | |
|  | Mato Grosso do Sul | Paranaíba | *Triatoma sordida* | Female | Peridomicile A | NEG | NEG | - | |
|  | Mato Grosso do Sul | Paranaíba | *Triatoma sordida* | Female | Peridomicile A | NEG | NEG | - | |
|  | Mato Grosso do Sul | Paranaíba | *Triatoma sordida* | Male | Peridomicile A | NEG | NEG | - | |
|  | Mato Grosso do Sul | Paranaíba | *Triatoma sordida* | Male | Peridomicile B | NEG | NEG | - | |
|  | Mato Grosso do Sul | Paranaíba | *Triatoma sordida* | Female | Peridomicile B | NEG | NEG | - | |
|  | Mato Grosso do Sul | Paranaíba | *Triatoma sordida* | Female | Peridomicile B | NEG | NEG | - | |
|  | Mato Grosso do Sul | Paranaíba | *Triatoma sordida* | Female | Peridomicile B | NEG | NEG | - | |
|  | Mato Grosso do Sul | Paranaíba | *Triatoma sordida* | Male | Peridomicile B | NEG | NEG | - | |
|  | Mato Grosso do Sul | Paranaíba | *Triatoma sordida* | Male | Peridomicile B | NEG | NEG | - | |
|  | São Paulo | Espírito Santo do Pinhal | *Triatoma wygodzinskyi* | Male | Sylvatic A | NEG | NEG | - | |
|  | São Paulo | Espírito Santo do Pinhal | *Triatoma wygodzinskyi* | N5 | Sylvatic A | NEG | NEG | - | |
|  | São Paulo | Espírito Santo do Pinhal | *Triatoma wygodzinskyi* | N5 | Sylvatic A | NEG | NEG | - | |
|  | São Paulo | Espírito Santo do Pinhal | *Triatoma wygodzinskyi* | N4 | Sylvatic A | NEG | NEG | - | |
|  | São Paulo | Espírito Santo do Pinhal | *Triatoma wygodzinskyi* | N4 | Sylvatic A | NEG | NEG | - | |
|  | São Paulo | Espírito Santo do Pinhal | *Triatoma wygodzinskyi* | N1 | Sylvatic A | NEG | - | - | |
|  | São Paulo | Espírito Santo do Pinhal | *Triatoma wygodzinskyi* | N2 | Sylvatic A | NEG | - | - | |
|  | São Paulo | Espírito Santo do Pinhal | *Triatoma wygodzinskyi* | N2 | Sylvatic A | NEG | - | - | |
|  | São Paulo | Espírito Santo do Pinhal | *Triatoma wygodzinskyi* | N2 | Sylvatic A | NEG | - | - | |
|  | São Paulo | Espírito Santo do Pinhal | *Triatoma wygodzinskyi* | N2 | Sylvatic A | NEG | - | - | |
|  | São Paulo | Vargem Grande do Sul | *Triatoma wygodzinskyi* | Female | Sylvatic A | NEG | NEG | - | |
|  | São Paulo | Vargem Grande do Sul | *Triatoma wygodzinskyi* | N5 | Sylvatic A | NEG | NEG | - | |
|  | São Paulo | Vargem Grande do Sul | *Triatoma wygodzinskyi* | N4 | Sylvatic A | NEG | NEG | - | |
|  | São Paulo | Vargem Grande do Sul | *Triatoma wygodzinskyi* | N4 | Sylvatic A | NEG | NEG | - | |
|  | São Paulo | Vargem Grande do Sul | *Triatoma wygodzinskyi* | N4 | Sylvatic A | NEG | NEG | - | |
|  | São Paulo | Vargem Grande do Sul | *Triatoma wygodzinskyi* | N4 | Sylvatic A | NEG | NEG | - | |
|  | São Paulo | Vargem Grande do Sul | *Triatoma wygodzinskyi* | N4 | Sylvatic A | NEG | NEG | - | |
|  | Rio de Janeiro | São Fidélis | *Triatoma vitticeps* | Female | Peridomicile A | NEG | NEG | - | |
|  | Rio de Janeiro | São Fidélis | *Triatoma vitticeps* | Female | Peridomicile A | NEG | NEG | - | |
|  | Rio de Janeiro | Santa Maria Madalena | *Triatoma vitticeps* | Female | Peridomicile A | **POS** | NEG | 3.71E+05 | |
|  | Rio de Janeiro | Santa Maria Madalena | *Triatoma vitticeps* | Female | Peridomicile A | **POS** | NEG | Not detected | |
|  | Rio de Janeiro | Tanguá | *Triatoma vitticeps* | Male | Peridomicile A | NEG | NEG | - | |
|  | Rio de Janeiro | Tanguá | *Triatoma vitticeps* | Male | Peridomicile A | NEG | NEG | - | |
|  | Rio de Janeiro | Tanguá | *Triatoma vitticeps* | Female | Peridomicile A | NEG | NEG | - | |
|  | Minas Gerais | Santa Rita de Caldas | *Triatoma wygodzinskyi* | N2 | Sylvatic A | NEG | - | - | |
|  | Minas Gerais | Santa Rita de Caldas | *Triatoma wygodzinskyi* | N2 | Sylvatic A | NEG | - | - | |
|  | Minas Gerais | Santa Rita de Caldas | *Triatoma wygodzinskyi* | N2 | Sylvatic A | NEG | - | - | |
|  | Minas Gerais | Santa Rita de Caldas | *Triatoma wygodzinskyi* | N2 | Sylvatic A | NEG | - | - | |
|  | Minas Gerais | Santa Rita de Caldas | *Triatoma wygodzinskyi* | N2 | Sylvatic A | NEG | - | - | |

*Abbreviations*: N1-N5, first to fifth triatomine instars; POS, positive for *T*. *cruzi* detection; NEG, negative for *T*. *cruzi* detection; –, not performed
